# Non-alcoholic fatty liver disease across endocrinopathies: Interaction with sex hormones

**DOI:** 10.3389/fendo.2022.1032361

**Published:** 2022-11-07

**Authors:** Sara Arefhosseini, Mehrangiz Ebrahimi-Mameghani, Farzad Najafipour, Helda Tutunchi

**Affiliations:** ^1^ Student Research Committee, Department of Biochemistry and Diet Therapy, Faculty of Nutrition and Food Sciences, Tabriz University of Medical Sciences, Tabriz, Iran; ^2^ Nutrition Research Center, Department of Biochemistry and Diet Therapy, Faculty of Nutrition and Food Sciences, Tabriz University of Medical Sciences, Tabriz, Iran; ^3^ Endocrine Research Center, Tabriz University of Medical Sciences, Tabriz, Iran

**Keywords:** growth hormone deficiency (GHD), nonalcoholic fatty liver disease (NAFLD), polycystic ovarian syndrome (PCOS), hypogonadism, hypopituitarism

## Abstract

Nonalcoholic fatty liver disease (NAFLD) has emerged as the most frequent chronic liver disease globally. NAFLD is strongly associated with metabolic syndrome and it has been recently suggested that to rename NAFLD as metabolic dysfunction-associated fatty liver disease (MAFLD). NAFLD has been studied in different endocrine axes and accumulating body of clinical and experimental studies have suggested that NAFLD is associated with polycystic ovarian syndrome (PCOS), hypopituitarism, growth hormone deficiency (GHD), hypogonadism and other endocrine disorders. In fact, endocrine dysfunction may be considered as the major contributor for the development, progression, and severity of NAFLD. In the present comprehensive review, we discussed the epidemiological and clinical evidence on the epidemiology, pathophysiology, and management of NAFLD in endocrine disorders, with an emphasis on the effects of sex-specific hormones/conditions as well as molecular basis of NAFLD development in these endocrine diseases.

## 1 Introduction

The liver, a dynamic endocrine organ, displays a crosstalk with extra-hepatic organs in order to mediate numerous metabolic, endocrine and hormonal signaling pathways (1). Non-alcoholic fatty liver disease (NAFLD) is the most common liver disease and is associated with a wide spectrum of hepatic disorders ranging from simple steatosis to nonalcoholic steatohepatitis (NASH), cirrhosis and hepatocellular carcinoma (HCC) (2). It is estimated that NAFLD may affect almost one third of the general population by 2030 (3). The “Multiple-Hit” theory, based on the synergic role of genetic and epigenetic factors including insulin resistance (IR), inflammation, oxidative stress (OS) and gut dysbiosis has been suggested for disease pathogenesis (4). Based on the metabolic roots of NAFLD, it has been recently suggested that to rename NAFLD as metabolic dysfunction-associated fatty liver disease (MAFLD) (5). Moreover, considering NAFLD as the “barometer of metabolic health” highlights the importance of metabolic disorders and associated endocrine dysfunction during disease progression (1, 6). NAFLD has been studied in different endocrine axes and to our knowledge, endocrinopathies are potent factors contributing to the incidence, progression and severity of NAFLD (7). Recently, accumulating body of clinical and experimental studies have suggested the association of NAFLD with polycystic ovarian syndrome (PCOS), hypopituitarism, growth hormone deficiency (GHD), hypogonadism and other endocrine disorders. Despite the extensive research that has been carried out, the exact inter-relationship between NAFLD and these conditions are not fully understood. In this review, we aimed to make an overview and summarize the epidemiologic, pathophysiologic and molecular approaches of recently published data on the association between NAFLD and endocrine disorders with an emphasize on the effects of sex-specific hormones/conditions.

## 2 Polycystic ovary syndrome

NAFLD-associated endocrinopathies, may be clinically displayed as other pathological conditions such as sex hormone imbalance. PCOS-a multifaceted disorder with a spectrum of clinical presentations- is known as the most prevalent endocrine disease in women of childbearing age (8). PCOS is a potent risk factor for anovulatory infertility with a prevalence of 5-20% worldwide based on the used diagnostic criteria (9). More precisely, disease prevalence has been reported 6-10% based on the classic definition, while, the number reaches to 18-20% by considering the Rotterdam criteria due to comprising meddler phenotypes in the absence of hyperandrogenism (HA) (10). Current diagnostic characteristics of PCOS include chronic ovulatory dysfunction, clinical and/or biochemical HA and polycystic ovarian morphology confirmed through ultrasonography (8). PCOS is diagnosed when at least two of the mentioned criteria are present after excluding other endocrine conditions (8). PCOS is a multifactorial disorder with a combination of metabolic, endocrinological, and genetic disturbances (8). Regarding the Rotterdam criteria, PCOS is classified into three phenotypes including “classical”, “ovulatory” and “normo-androgenic” with different risks for the development of metabolic disorders (11). The women in “classical” subgroup exhibit the highest risk of IR and metabolic syndrome (Mets) in comparison to others (11). In this context, PCOS is known as a multi-system disease due to its concomitance with other disorders such as Mets, type 2 diabetes mellitus (T2DM), cardiovascular- and cerebrovascular –diseases (10). Indeed, obesity is an additional finding in almost 40% of PCOS patients, while it has been eliminated from disease diagnostic features (11). Obesity, in turn, may also lead to IR with subsequent hyperinsulinemia as an innate feature (11). Moreover, a number of studies imply that women with PCOS experience higher IR compared with healthy subjects, independent of body mass index (BMI) status (12). Given that up to 70% of patients with PCOS are diagnosed with IR, it is considered as one of the focal pathogenic pathways linking PCOS to other metabolic and endocrine disorders (13). Taken together, IR, obesity, metabolic disorder and androgen excess commonly trigger the risk of NAFLD among these patients at the baseline or over the time (14).

### 2.1 Epidemiology of NAFLD in PCOS

To date, NAFLD is a growing global health problem with a great concern for the health-care system, highlighting the need for exploring high-risk populations and managing related outcomes. NAFLD and PCOS are not only co-exist conditions, but also show a synergic burden based on their common pathological pathways (15). Moreover, the emerging rapid increase in the prevalence of NAFLD and PCOS shows the importance of conducting related epidemiological studies. The role of PCOS in NAFLD progression seems to remain unclear. To the best of our knowledge, while a great number of studies have documented the significant increased prevalence of NAFLD (generally in ultrasound-proven subjects) in patients with PCOS, other results remained inconclusive (16). For the assessment, PCOS was diagnosed through National Institutes of Health (NIH) 1990 definition, Rotterdam 2003 criteria, or Androgen Excess Society criteria (17). Furthermore, fatty liver was detected *via* assessing serum liver enzymes, ultrasonography, transient elastography, magnetic resonance spectroscopy (MRS) and liver biopsy. Brown et al. (18) first hypothesized a relation between PCOS and NAFLD in a case report in a 24-years old woman with PCOS and IR who was assessed for NAFLD *via* liver biopsy. Regarding to the literature review, four previous meta-analyses have investigated the interaction between NAFLD and PCOS. Ramezani-Binabaj et al. (19) first performed a meta-analysis of 7 studies in 2014 and reported the significant higher prevalence of NAFLD in women with PCOS as well as introducing PCOS as a probable risk factor for NAFLD progression. Due to the limitation of included studies, Rocha et al. (20) with regard to the collected data from 17 studies of 2734 cases vs. 2561 age and BMI-matched controls, confirmed previous results beside assessing the levels of serum androgen. In this meta-analysis, the higher prevalence of NAFLD among PCOS patients was attributed to increased free androgen index (FAI), serum total testosterone (TT), BMI and IR (20). Moreover, serum androgens were known as independent risk factors for NAFLD in PCOS patients by considering the finding that normo-androgen-PCOS women had normal liver function indices in comparison to PCOS women with HA (21). Subsequently, another meta-analysis in 2018 was conducted on 17 observational studies to clarify whether PCOS acts as an independent trigger for NAFLD or their co-existence is related to their mutual risk factors (22). Results were in accordance with former studies. Furthermore, after stratification for BMI and geographic origin, the marked higher risk of NAFLD was reported in PCOS women with HA (22). Wu et al. (22) shed a light into the possible correlation between androgen levels and NAFLD in an independent manner of IR. The most recent updated meta-analysis in 2021 with a larger sample size was carried out by Shengir et al. (15) including 23 studies with 7148 participants (4164 PCOS women vs 2984 controls) in agreement with previous meta-analyses. In all, aforementioned studies suggest that while liver biopsy is the gold-standard diagnostic method, the majority of studies have used ultrasonography for assessing NAFLD due to its reproducibility, low cost and noninvasiveness as well as satisfactory sensitivity and specificity for epidemiologic studies. Nevertheless, a small group of studies have used liver enzymes, biopsy and other diagnostic methods. As mentioned by Rocha et al. (20), the results seem to indicate an increased risk of NAFLD in PCOS suggesting that the heterogeneity in study designs and screening tools did not affect the overall result.

Based on the previous reports, contributing factors in NAFLD progression among PCOS women include IR, HA and disturbed metabolic markers, meanwhile, the current study depicts BMI as the main cofactor (15). A number of observational studies assessed the correlation between higher serum liver enzymes and PCOS (23–27). In this context, Schwimmer et al. (25) reported the higher risk of elevated serum alanine transaminase (ALT) in PCOS subjects after adjusting for confounders (30% and 15% increased risk for > 35 U/L and > 60 U/L cut-off values, respectively). The correlation between PCOS and increased serum aminotransferases was also observed in adolescent PCOS patients with a 15.4% prevalence (28). Two main factors, namely age and TT, have been reported to accelerate the progression of liver steatosis in adolescents (29). Taken together, current evidence demonstrates a positive correlation between increased aminotransferase levels and PCOS, although reported variations in prevalence may be related to study characteristics and performed diagnostic criteria (13). Besides, ALT levels have been attributed to lower insulin sensitivity in PCOS women (30). Other studies investigated the epi-phenomenon by using ultrasound data for detecting NAFLD (19, 23, 31–33). Gambarin-Gelwan et al. (12) demonstrated a 55% prevalence of NAFLD among PCOS women *via* ultrasonography. Interestingly 39% of the NAFLD proven subjects were lean. However, other studies did not report any significant correlation between ALT levels and PCOS in lean subgroup (34, 35). According to the lower prevalence of PCOS in lean women, the correlation between NAFLD and PCOS is still doubted in lean subjects (36). Compensatory, case- control studies also were performed to assess the issue. A study by Cerda et al. (23) showed higher prevalence of ultrasound confirmed-NAFLD (41.5% vs 19.4%, respectively) and elevated ALT levels (39% and 3.2%, respectively) in patients with PCOS in comparison to healthy controls. Moreover, ultrasound findings have illustrated increased thickness of mesenteric fat in patients with NAFLD and PCOS, suggesting an independent risk factor in these patients (33). Latter investigations used transient elastography as another non-invasive tool for the assessment and conducted cross-sectional studies in South Asia and Mexico reported similar results that represent the significant higher risk (37, 38). MRS was also used to detect intra-hepatic fat content and showed similar findings (21). Furthermore, other studies considered liver biopsy as the gold standard for the detection of steatosis (24, 39). Setji et al. (24) for the first time investigated histological aspects of biopsy-proven liver fibrosis and PCOS co-incidence. Based on their results, 15% of the PCOS patients experienced higher aminotransferase levels. Moreover, all of the patients with permanent increased ALT levels showed liver fibrosis signs through liver biopsy (24). Hence, increased risk of advanced NASH and liver fibrosis is anticipated in PCOS (40). Two further cross-sectional studies conducted in tertiary gastroenterology centers confirmed the presence of PCOS in 51%-71% of biopsy-proven NASH patients (39, 41). Limited number of studies have also evaluated liver steatosis/fibrosis-related indices in PCOS populations. In this regard, Polyzos et al. (42) revealed a positive correlation between fatty liver index (FLI), lipid accumulation products (LAP), hepatic steatosis index (HSI) and AST to platelet ratio (APRI) with PCOS (27, 42). A most recent large scale cross-sectional study from US national database, containing data from 50,785,354 women including 77,415 PCOS patients demonstrated a 4.3- fold higher prevalence of NAFLD in PCOS subjects, after adjusting for confounders (43). They also considered HA as the potential link in this epi-phenomenon (43). In this line, a great number of studies were performed to assess the predictors of NAFLD among PCOS patients. Collectively, the predictors include obesity, Mets, IR and HA. To the best of our knowledge a majority of studies have suggested androgen excess as the main risk factor for co-existence of PCOS and NAFLD (37, 38, 44, 45). The “classical phenotype” of PCOS exhibited a marked higher prevalence of NAFLD in comparison to other phenotypes (84.3% vs 41.1%, respectively) (38). Hence, HA has been considered as a putative risk factor solely (21, 31, 37, 46, 47) or along with increased BMI (37) or IR (45). In this regard, a large scale retrospective cohort study was conducted by Kumarendran et al. (48) and the raised incidence of NAFLD among PCOS women was attributed to HA, BMI and dysglycemia after assessing 63,000 PCOS women vs 121,000 controls. The latest study by Won et al. (44) involved 586 PCOS subjects and highlighted HA and Mets as the main related risk factors for the incidence of NAFLD in these patients. Although meta-analyses confirmed mentioned data and introduced serum androgens as independent predictors of NAFLD in PCOS women, there are a few number of findings making this issue controversial (20). Two studies did not observe any marked association between sex hormone-binding globulin (SHBG), FAI, TT and 17-hydroxyprogesterone levels with NAFLD (49). Overall, some studies have reported the correlation between FAI, SHBG and TT with NAFLD (26, 31, 35, 41, 50, 51) while other studies have failed to confirm these associations (12, 35, 49, 52–54). BMI, Mets, metabolic disturbances (e.g. higher serum triglyceride (TG), LAP) are among other important factors linking two diseases (15, 53). In addition, limited studies have suggested a number of other mutual risk factors including exacerbating inflammatory status and menstrual/reproductive markers, in an overweight-obesity-independent manner (47). The results of studies on risk factors for development of NAFLD in PCOS patients are summarized in **Table 1**.

**Table 1:** Studies investigating the risk factors for the development of NAFLD in PCOS patients.

Studies	Study design	Study population	Risk factors	Findings
Brown et al (2005) (18)	Case report	A 24 years old woman (BMI: 37 Kg/m^2^)	IR and metabolic disturbances	Link between NAFLD and PCOS
Schwimmer et al. (2005) (25)	Retrospective, cross-sectional	Country: USA 70 PCOS patients (Mean age: 28 yr., varied BMI)	IR, obesity and metabolic disturbances	Higher risk of abnormal ALT in women with PCOS
Setji et al (2006) (24)	Retrospective chart review	Country: North Carolina Multi- ethnic 200 PCOS patients (Age: 18-50 yr., BMI>35 Kg/m^2^)	TG, AST, ALT, fasting insulin and lower HDL	Higher risk of advanced stage of NASH seen on the biopsies of PCOS patients
Gambarin- Gelwan et al (2007) (12)	Retrospective, cross-sectional	Multi- ethnic 88 overweight premenopausal PCOS patients (Mean age: 31.4 yr., varied BMI)	BMI, HOMA-IR and lower HDL	Higher risk of NAFLD in patients with PCOS (55%) with normal serum liver enzymes in most of them
Barfield et al (2009) (28)	Retrospective chart review	Multi- ethnic 39 obese adolescent with PCOS	Liver dysfunction and Mets	Higher prevalence of serum aminotransferase levels (15.4%) in PCOS adolescents
Brzozowska et al (2009) (41)	Pilot, cross-sectional	Country: Australia 14 obese, biopsy/sonography- NAFLD confirmed women (Age: 20-45 yr., BMI:28-40 Kg/m^2^)	BMI and metabolic disturbances	Higher prevalence of PCOS in patients with NAFLD, and evidence regarding to advanced liver disease in PCOS patients
Economou et al (2009) (35)	Case- control	Country: UK PCOS patients (n=83) *vs* controls (n=64) (Mean age: 25 yr., varied BMI)	Fasting insulin, FBS, HOMA-IR, TG and TC	Significant higher serum ALT and GGT just in overweight/obese PCOS women and insignificant differences in lean subjects
Tan et al (2009) (55)	Case- control	Country: Germany PCOS patients (n=192) *vs* controls (n=73) (Mean age: 29 yr., Mean BMI= 31.5 Kg/m^2^)	Higher M30 levels, LDL, BMI, FAI and lower HDL	Significant increased M30 levels in PCOS women as the apoptotic marker related to NASH progression
Targher et al (2009) (30)	Case- control	Country: Italy PCOS patients (n=14) PCOS patients with NAFLD (n=14) Controls (n=14) (Mean age: 23 yr., Mean BMI: 23.7 Kg/m^2^)	Decreased insulin sensitivity and HDL with increased TG	Higher ALT levels related to markedly decreased insulin sensitivity in PCOS women
Kauffman et al (2010) (52)	Retrospective pilot	Country: USA PCOS patients (n=32) PCOS patients with NAFLD (n=21) (Age: 18-42 yr., BMI >25 Kg/m^2^)	IR	IR as the linkage between PCOS and NAFLD (no difference in sex hormones)
Ma et al (2011) (33)	Cross-sectional	Country: Hong Kong 117 PCOS patients (Mean age: 28.6 yr., Mean BMI: 24.3 Kg/m^2^)	Fasting insulin and mesenteric fat thickness	Mesenteric fat as an independent risk factor of NAFLD in PCOS and increased cardiovascular risk
Jones et al (2012) (21)	Case- control	Country: UK Young PCOS patients (n = 29) vs controls (n = 22) (Mean age: 28 yr., Mean BMI: 33 Kg/m^2^)	HA	Higher intra-hepatic fat content in PCOS women with HA
Michaliszyn et al et al (2013) (29)	Cross-sectional case–control	Multi- racial 30 obese adolescent PCOS patients (Mean age: 16.1 yr., Mean BMI: 37.1 Kg/m^2^)	Age and total testosterone	No difference in aminotransferase levels between groups, metabolic changes and NAFLD with increasing age
Tarantino et al (2013) (56)	Cross-sectional Case-control	Country: Italy, Caucasian Premenopausal PCOS patients (n=60) Vs controls (n=20) (Age: 15-40 yr., BMI: 18-46 Kg/m^2^)	IR and spleen size	Role of spleen-liver axis and IR in co-incidence of NAFLD in PCOS
Bohdanowicz -Pawlak et al (2014) (46)	Case- control	Country: Poland PCOS patients (n=184) vs Controls (n=125) (Mean age: 25.3 yr., BMI >25 Kg/m^2^)	HA	High risk of NAFLD in women with HA by inducing hepatotoxicity and not *via* obesity and IR
Dawson et al (2014) (49)	Cross-sectional	25 patients: PCOS without NAFLD (n=12) PCOS with NAFLD (n=13) (Mean age: 28 yr., BMI>30 Kg/m^2^)	BMI and WC	Greater BMI without differences in inflammatory or endothelial and hormonal markers in PCOS patients with NAFLD
Polyzos et al (2014) (42)	Cross- sectional	Country: Greece PCOS patients (n=314) PCOS (n=237) PCOS + Mets (n=77) Controls (n=78) (Age>25 yr., BMI >25 Kg/m^2^)	Mets	Higher liver steatosis-related indices in PCOS group
Ramezani- Binabaj et al (2014) (19)	Meta- analysis	7 studies (2007-2013) PCOS patients (n=616) vs Controls (n=569) (Mean age: 27 yr., Mean BMI: 29 Kg/m^2^)	IR and Mets	Higher risk of NAFLD in patients with PCOS
Rocha et al (2017) (20)	Meta- analysis	17 studies (2007-2017) PCOS patients (n=2734) vs Controls (n=2561) (Age: 17-38 yr., BMI: 20-45 Kg/m^2^)	HA, IR and obesity	Higher prevalence of NAFLD in PCOS patients markedly in classic phenotype
Kumarendran et al (2018) (48)	Population-based retrospective cohort	Country: UK PCOS patients (n=63,120) vs controls (n=121,064) from United Kingdom primary care database (Mean age: 30 yr., varied BMI)	HA, BMI and dysglycemia	Higher rate of NAFLD in patients with PCOS
Wu et al (2018) (22)	Meta- analysis	17 studies (2007-2017) PCOS patients (n=2715) vs matched controls (n=2619) (Varied age and BMI)	HA	High risk of NAFLD among PCOS patients, especially women with HA
Varma et al (2019) (45)	Prospective, cross Sectional	Asian Indian 60 PCOS patients (Mean age: 24.06 yr., Mean BMI: 29.5 Kg/m^2^)	HOMA-IR and HA	Increased prevalence of NAFLD in PCOS patients (38.3%)
Asfari et al (2020) (43)	National US Inpatient Sample Database (2002-2014)	Multi- racial Total of 50785354 women 77415 PCOS patients (Age>18 yr., varied BMI)	HA	Higher NAFLD prevalence in PCOS patients (p<0.001)
Salva- Pastor Et al (2020) (38)	Cross- sectional	Country: Mexico 98 PCOS patients vs (1:1) Controls (Age: 18-44 yr., BMI>25 Kg/m^2^)	BMI and HA	NAFLD prevalence in PCOS patients (69.3%), especially phenotype A (84.3%) vs controls (34.6%)
Sarkar et al (2020) (40)	Retrospective	Country: USA, Multi- racial 102 biopsy-proven NAFLD Women (Age: 18-45 yr., BMI>30 Kg/m^2^)	Higher risk of severe fibrosis and hepatocyte ballooning following PCOS	PCOS prevalence in NAFLD patients (36%)
Shengir et al (2020) (37)	Cross- sectional cohort	Country: South Asia 101 PCOS patients (Mean age: 36.3 yr., BMI> 25 Kg/m^2^)	BMI, ALT and HA	NAFLD prevalence (39.6%) liver fibrosis (6.9%)
Wang et al (2020) (47)	Case series	Country: China PCOS patients (n=501) Controls (n=112) (Mean age: 26 yr., BMI: 20-31 Kg/m^2^)	Androgen levels	Induction of NAFLD by androgen levels *via* affecting liver fat content in a dimorphic pattern
Shengir et al (2021) (15)	Meta- analysis	23 studies (2007-2019) PCOS patients (n=4164) vs Controls (n=2984) were matched (Age: 25-33 yr., BMI: 21-34 Kg/m^2^)	BMI	A 2.5-fold increase in NAFLD in PCOS patients compared to controls
Won et al (2021) (44)	Retrospective cohort	Country: Korea 586 PCOS patients (Age: 13-35 yr., BMI>25 Kg/m^2^)	Mets and HA	NAFLD prevalence in PCOS patients (8.7%)

ALT, Alanine transaminase; AST, Aspartate aminotransferase; BMI, Body mass index; FAI, Free androgen index; FBS, Fasting blood sugar; GGT, gamma- glutamyl transferase; HA, Hyperandrogenism; HDL, High-density lipoprotein cholesterol; HOMA-IR, Homeostatic model assessment of insulin resistance; IR, Insulin resistance; LDL, Low-density lipoprotein cholesterol; Mets, Metabolic syndrome; NAFLD, Non-alcoholic fatty liver disease; NASH, Non-alcoholic steatohepatitis; PCOS, Polycystic ovarian syndrome; TC, Total cholesterol; TG, Triglycerides.

### 2.2 Pathophysiology of NAFLD in PCOS

**Figure 1:**
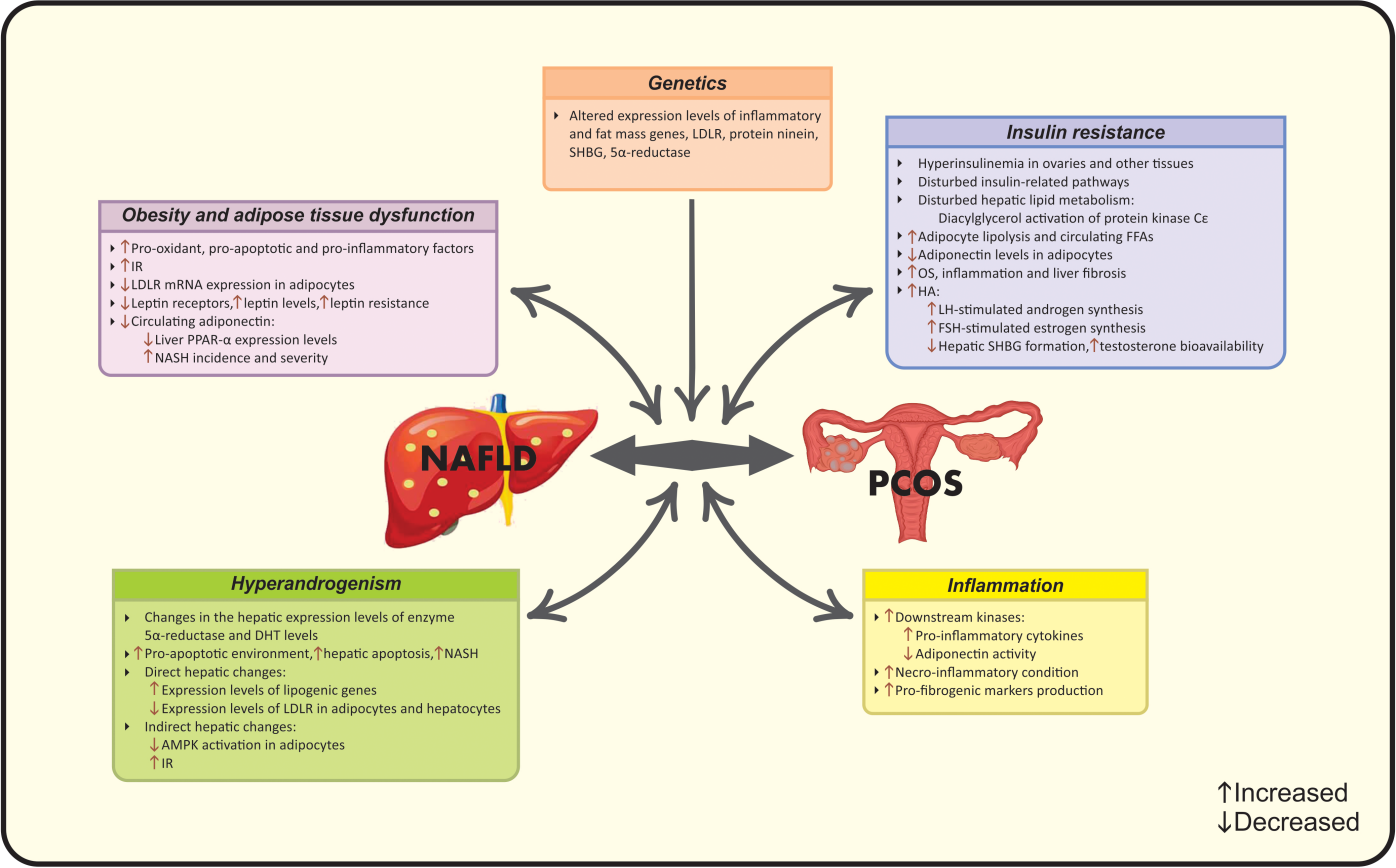
Established pathophysiological mechanisms linking PCOS and NAFLD. AMPK, Adenosine monophosphate-activated protein kinase; DHT, Dihydrotestosterone; FFA, Free fatty acid; FSH, follicle-stimulating hormone; HA, Hyperandrogenism; IR, Insulin resistance; LDLR, Low-density lipoprotein receptor; LH, Luteinizing hormone; NAFLD, Nonalcoholic fatty liver disease; NASH, Non-alcoholic steatohepatitis; OS, Oxidative stress; PCOS, Polycystic ovarian syndrome; PPARα, Peroxisome proliferator-activated receptor-α; SHBG, Sex hormone-binding globulin.

**Figure 2:**
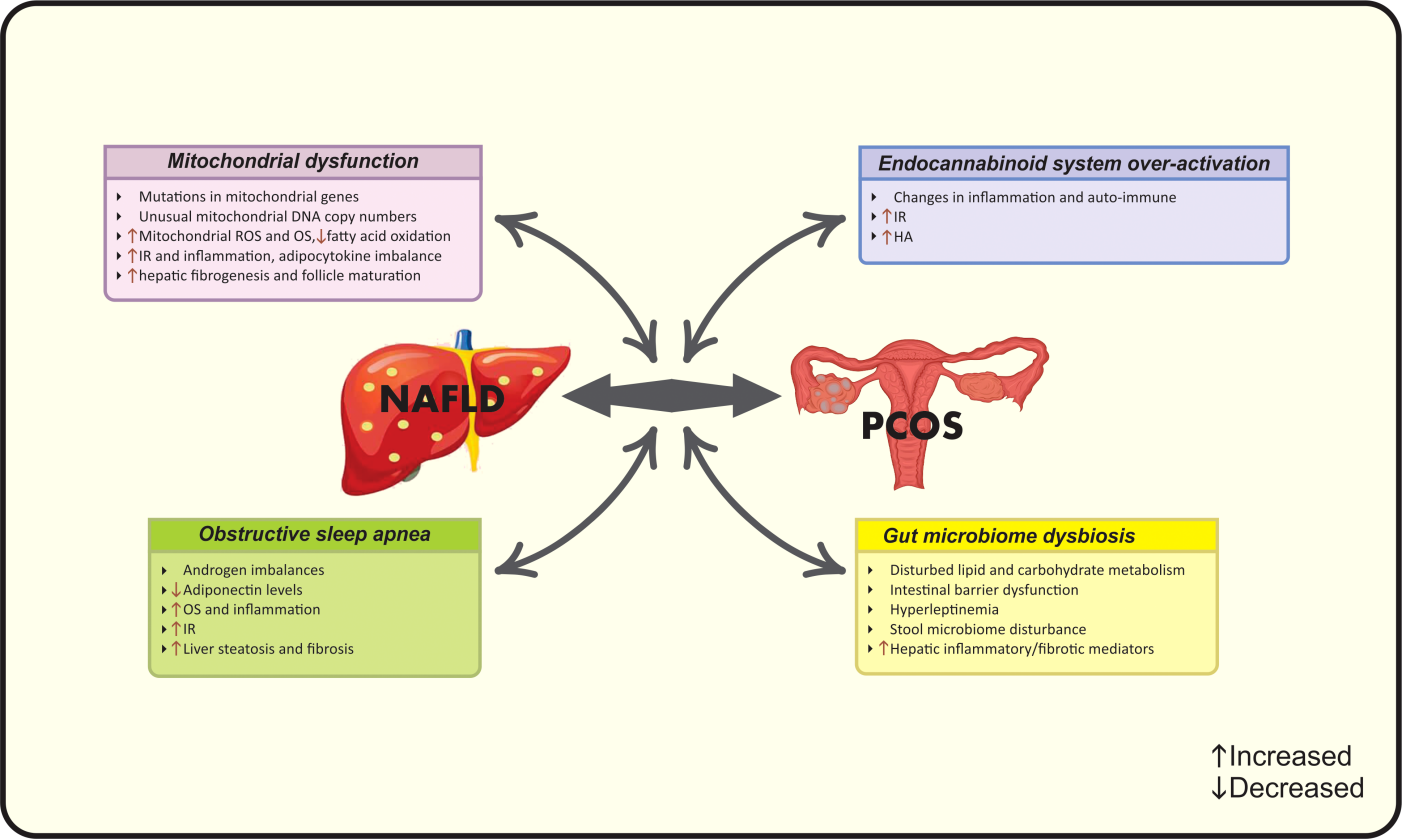
Possible new pathophysiological mechanisms linking PCOS and NAFLD. HA, Hyperandrogenism; IR, Insulin resistance; NAFLD, Nonalcoholic fatty liver disease; OS, Oxidative stress; PCOS, Polycystic ovarian syndrome; ROS, Reactive oxygen species.

In the light of the controversy among current epidemiological findings, a number of studies suggest the correlation between NAFLD and PCOS, while others do not (15). Meanwhile, to date, there are four met-analysis studies that document higher risk of NAFLD in PCOS women *via* their common risk factors including obesity (19), BMI (15), IR (19), HA (20) and metabolic factors (15). Hence, it seems that a multifactorial pathological mechanism- as the complex of genetic and acquired factors- contributes to this co-existence (16). A vicious cycle of genetic variations, adipocyte- dysfunction, HA, systemic IR and chronic inflammation contribute to the synergistic catastrophic consequence of aforementioned diseases (16). Evidence seems to be insufficient for determining whether the association between NAFLD and PCOS is resulted from their common risk factors or PCOS acts as an independent risk factor for NAFLD. Different mechanisms linking PCOS and NAFLD are summarized in **Figures 1** and **2**.

#### 2.2.1 Obesity and adipose tissue dysfunction

Abdominal obesity is another common finding in both NAFLD and PCOS patients and at the same time is associated with other related mutual risk factors (17). The term “hypertrophic obesity” used for PCOS women refers to the larger adipocytes (shown as its 25% increased diameter compared with healthy obese controls) as well as higher amounts of visceral fat, markedly in mesenteric and intraperitoneal sites (57). Villa et al. (57) reported that although this specific pattern of fat distribution in PCOS patients is not a putative factor in disease progression, the adipose tissue dysfunction is linked to several metabolic abnormalities. Several observational studies have introduced obesity as a common mechanism linking NAFLD and PCOS (58, 59). Noteworthy, the impaired visceral adipose tissue becomes a target for systemic alterations and pathologic mediators such as pro-oxidant, pro-apoptotic and pro-inflammatory factors and thereby significant metabolic/hormonal disorders emerge as the co-existence of endocrine-related diseases such as PCOS and NAFLD (16). IR, HA and apoptosis are the main contributors. In addition to IR, which is a characteristic feature of hypertrophic adipocytes, HA induces IR in visceral adipocytes and deteriorates abdominal adiposity in PCOS women (57). Another androgen-dependent change refers to increased serum M30 levels-as an apoptosis biomarker- along with decreased low-density lipoprotein receptor (LDLR) mRNA expression in adipocytes of PCOS women with NAFLD compared with NAFLD patients that sheds a light into the importance of adipocyte dysfunction (59).

#### 2.2.2 Insulin resistance

Based on the clinical evidence, IR is present in 80% of patients with NAFLD and 50-80% of patients with PCOS plus NAFLD (60). IR plays an important role in the development of NAFLD and PCOS (60). The severity of both diseases is associated with higher IR (30). IR may be a consequence of obesity or lowered glucose uptake in muscles followed by increased hepatic *de novo* lipogenesis (10). The accumulation of hepatic lipotoxic products reduces mitochondrial fatty acid oxidation and manifests as IR in NAFLD (61). IR, in turn, is linked with a wide spectrum of adverse conditions such as OS, inflammation, adipocytokine imbalance and liver fibrosis (56, 61). IR is also associated with increased adipocyte lipolysis and higher levels of free fatty acids in circulation (38). In the case of PCOS, impaired insulin signaling, due to the post-receptor defects in insulin signal transduction in adipocytes and muscles, leads to the additional hyperinsulinemic condition in other tissues such as the ovaries (61). Finally, these changes emerge as the IR-phenotype in women with PCOS which make changes in androgenesis (10). Insulin acts as a co-gonadotropin to increase luteinizing hormone (LH)-stimulated androgen synthesis and follicle-stimulating hormone (FSH)-stimulated estrogen synthesis as well as to decrease hepatic formation of SHBG which is the key binding protein for testosterone and prolongs its metabolic clearance, leading to enhanced testosterone bioavailability (13). This androgen-regulating aspect of insulin is amplified during the hyperinsulinemic state of PCOS, especially in theca cells (61). Hence, insulin can regulate the generation, bioavailability and clearance of androgens and make androgen excess state (13). Androgen excess in turn may lead to IR by lowering adiponectin levels in adipocytes that is followed by enhanced visceral adiposity and disturbed insulin action (62).

#### 2.2.3 Hyperandrogenism

PCOS is already known as an outstanding “heyperandrogenic syndrome”, encompassing a wide range of changes in endocrine and metabolic markers (13). The majority of studies have investigated the androgen changes in PCOS along with NAFLD. It is considered that androgen levels contribute to the development of NAFLD in a sex-dependent manner i.e. hypogonadism in men and hyperandrogenism in women (63). In the concept of “hepato-ovarian axis”, liver is known as the origin and the target of hormonal changes at the same time. Two pathways are involved; hepatic involvement in peripheral sex-steroid transformation beside the hepatic-surface expression of androgen-estrogen receptors (64). The presence of persist IR state along with lowered HA after the ovarian ablation surgery sheds a light into the issue that IR and HA are the main causes of the co-existence of NAFLD and PCOS, but there is a gap in the priority between them (25, 45). As mentioned before, IR results in HA-phenotype. Wang et al. (47) also confirmed that HA in turn contributes to the development of NAFLD. These mechanisms of action are *via* direct and/or indirect hepatic changes; direct effect of androgen excess leads to hepatic fat accumulation, while, indirect effects include (a) suppressing adenosine monophosphate-activated protein kinase (AMPK) activation in adipocytes that results in higher lipogenesis and increased visceral adiposity and (b) exerting alterations in insulin sensitivity or both mechanisms together (22, 65). Changes in insulin sensitivity have been observed as HA-induced-IR *via* the suppression of insulin signaling transduction (66). Aforementioned changes induce disturbances in lipid metabolism and emerge as adipose tissue dysfunction, collectively named as “androgen-mediated adipose lipotoxicity”, linking PCOS to NAFLD (67). HA leads to changes in the expression levels of genes involved in lipid metabolism represent in liver and adipocytes, thereby making PCOS subjects more susceptible to NAFLD (59). Kumarendan et al. (48) recently observed a correlation between higher free testosterone levels (>3.0 nmol/L) and increased prevalence of NAFLD in PCOS women. In this regard, the hepatocytes express the enzyme 5α-reductase, which is involved in the converting circulating testosterone to dihydrotestosterone (DHT) and, on the other hand, modulates insulin pathway, liver steatosis and fibrosis (68). Androgens also induce a pro-apoptotic environment in PCOS patients (59, 69). Tan et al. (55) demonstrated higher serum levels of “M30” for assessing caspase 3-cleaved fragment of cytokeratin 18 (CK18)- the apoptotic biomarkers- in PCOS women and its role in the prediction of NASH. Another study showing higher M30 levels in patients with PCOS and NAFLD (proved by biopsy), confirmed previous data and highlighted the increased risk of hepatic apoptosis and NASH in women with PCOS (59). These findings highlighted the independent role of androgen levels in NAFLD regardless of IR or obesity (31). In this context, a recent meta-analysis has confirmed that PCOS patients with HA exhibit higher incidence of NAFLD after adjusting for confounders, while patients without HA show similar features to healthy subjects (22). Interestingly, there are a few number of studies in contrast with these findings, providing the non-significant difference in NAFLD prevalence among PCOS patients with or without HA (12, 35, 49, 52–54, 70).

#### 2.2.4 Inflammation

Low-grade inflammatory state not only can be identified as an additional link between two pathological entities but also plays a pivotal role in the progression of them (16). Inflammatory mediators can lead to IR and thereby, facilitate the progression of NAFLD in a multi-step manner (16). Tumor necrosis factor-alpha (TNF-α) is an important pro-inflammatory biomarker involved in this epiphenomenon. It is reported that patients with IR exhibit higher circulating TNF-α levels in an obesity-independent manner. Moreover, increased expression levels of androgen receptors (ARs) up-regulates the expression of TNF-α (58, 71). Increased TNFα induces IR and triggers downstream kinases, leading to higher cytokine formation along with impaired adiponectin activity (71). Taken together, low-grade inflammation plays a fundamental role in IR, abdominal obesity and NAFLD development (14). More severe forms of NAFLD are more susceptible to PCOS, due to the necro-inflammatory state that leads to the overproduction of multiple pro-inflammatory and profibrogenic markers (16).

#### 2.2.5 Genetics

Genetic polymorphisms are responsible for observed inter-ethnic differences in the prevalence of NAFLD in PCOS patients. Genome-wide association studies (GWAS) aimed to identify the molecular and genetic pathways involved in pathological conditions (72). The first GWAS identifies susceptibility loci for PCOS on chromosome 2p16.3, 2p21 and 9q33.3 (72). Moreover, polymorphisms in pro-inflammatory cytokine genes (interleukins) are detectable in PCOS (73). GWAS on NAFLD documented a missense mutation in patatin-like phospholipase domain-containing (PNPLA) 3 gene that completely overshadows NAFLD spectrum (74). Bohdanowicz-pawlak et al. (46) did not document any association between polymorphisms rs328 and rs268 of the lipoprotein lipase gene and NAFLD occurrence in women with PCOS. Previous studies have identified alterations in the expression levels of genes involved in both NAFLD and PCOS including LDLR, protein ninein, fat mass gene, SHBG, 5-alpha reductase along with inflammatory genes (59, 60).

#### 2.2.6 Mitochondrial dysfunction

Newly, a body of evidence indicates the role of mitochondrial dysfunction in NAFLD-PCOS connection. Patients with PCOS have shown mutations in mitochondrial genes and unusual mitochondrial DNA copy numbers (75). Moreover, this condition has been observed in PCOS patients with normal ranges of BMI (76). Indeed, IR as well as hepatic fat accumulation results in mitochondrial dysfunction and reduced fatty acid oxidation (61). It is considered that increased production of mitochondrial reactive oxygen species (ROS) plays a key role in producing liver damage and initiating hepatic fibrogenesis (77). Mitochondrial dysfunction induces OS that subsequently aggravates lipid metabolism, IR, inflammation, adipocytokine imbalance, and follicle maturation leading to the coexistence of PCOS and NAFLD (61, 75).

#### 2.2.7 Gut microbiome dysbiosis

“Gut-liver axis” has a predominant role in the liver health. The intestinal factors such as dysbiosis and intestinal barrier dysfunction along with hepatic factors such as inflammatory/fibrotic mediators and hyperleptinemia lead to NAFLD progression through disturbed lipid and carbohydrate metabolism (78). On the other hand, changes in the gut microbiome also play a role in PCOS progression and are explained by the disturbances in stool microbiome associated with IR, leading to reproductive and metabolic dysfunction of PCOS women (79). Collectively, gut microbiome can be considered as a newly diagnosed link between PCOS and NAFLD.

#### 2.2.8 Endocannabinoid system over-activation

To date, over-activation of the endocannabinoid system (ECS) is also involved in both diseases. ECS- a complex cellular signaling network- modulates a wide range of human functions and regulates inflammation, auto-immune and energy homeostasis (80). ECS is linked with non-receptor mediated pathways including the induction of MAP kinases and PI3 kinases beside G protein-independent pathways (81). The ECS includes cannabinoid receptors (CB1 and CB2) and their lipidic ligands and over-activated ECS is involved in different pathological conditions such as liver disease (82). ECS over-activation exerts catastrophic effects on insulin signaling and leads to IR, increased hepatic lipogenesis, inflammation, steatosis and subsequent NAFLD (80). CB1 polymorphism leads to the development of NAFLD and PCOS by progressing HA state (83).

#### 2.2.9 Obstructive sleep apnea

A commonly observed condition in women with PCOS and NAFLD is obstructive sleep apnea (OSA). Subjects with OSA have a 2.6- fold increased risk of NAFLD and fibrosis progression, while this number increases to 7.6-fold in patients with PCOS and OSA (84). OSA is linked with androgen imbalances and IR through the role of chronic intermittent hypoxia that leads to decreased levels of adiponectin, increased OS and inflammatory state, which ultimately manifests as liver steatosis (85). Regarding to the role of androgens in OSA, PCOS women with higher free testosterone levels are more prone to mentioned conditions (84).

### 2.3 Mechanisms linking NAFLD to PCOS

As discussed below, the role of abnormalities in IR pathway during NAFLD and PCOS has been confirmed previously. Accumulating evidence illustrated the disturbances in the initial part of glycogen metabolism and/or insulin- signaling cascades shown in IR-phenotype of PCOS (86). Insulin stimulates P450c17 activity through phosphoinositide 3-kinase (PI3K) and mitogen-activated protein kinase (MAPK) pathways, consisting from subgroup mediators such as MAP kinase-activated protein kinase-3, mitogen-activated protein kinase/extracellular signal-regulated kinase (MEK/ERK) and MAP kinase-activated protein kinase-4/c-Jun N-terminal kinase (87). Hence, not only the direct effects of insulin on androgens happen, but also disturbances in insulin-related pathways (PI3K, MAPK and MEK/ERK) are involved in androgen excess pattern in the IR- related- PCOS and NAFLD (88). Moreover, a controversy has been observed according to the differences in the underlying mechanisms of lean PCOS subjects compared to overweight/obese ones. It is suggested that in overweight/obese women with PCOS, IR state is due to the impairments in the proximal part of insulin signaling pathway in skeletal muscles, whereas in lean subjects, adiponectin affects skeletal muscle through AMPK mediators (89). IR state-found in either NAFLD or PCOS- may be also derived from the diacylglycerol activation of protein kinase Cϵ that is stimulated *via* disturbed hepatic lipid metabolism during diseases (90). IR promotes the hyperandrogenic aspect of PCOS. Androgen excess is also involved in liver lipotoxicity, as suggested by the impact of higher TT levels on increasing the expression levels of lipogenic genes as well as hepatic *de novo* lipogenesis in hepatic cells of women (67). More precisely, androgen excess reduces the expression levels of LDLR mRNA in adipocytes and hepatocytes of PCOS women (50, 59). These changes may result in extended half-life of very low-density lipoprotein cholesterol (VLDL-C), LDL-C, increased hepatic fat accumulation and eventually liver steatosis (50, 59). Oppositely, estrogen inhibits stellate cell activation and fibrogenesis. Women with PCOS and NAFLD showed lower estradiol levels compared with PCOS ones (91). Yang et al. (92) reported the increased risk of severe hepatic fibrosis in men when compared with premenopausal women. Moreover, the severity of NAFLD correlates with estrogen reduction (93). These findings may be explained by the protective role of estrogen against fibrogenesis.

Although there is limited evidence with regard to the role of adipokines in the mentioned co-existence, it is hypothesized that decreased number of leptin receptors may result in leptin resistance and increased leptin levels (94). Conversely, lowered circulating adiponectin is documented in PCOS women and NAFLD patients, respectively (95, 96). Meanwhile, Baranova et al. (60) did not find any significant variations according to adiponectin levels in NAFLD patients with or without PCOS. The underlying mechanism is based on the positive correlation between serum adiponectin levels and liver peroxisome proliferator-activated receptor-α (PPAR-α) mRNA expression level that exerts an inverse relation with NASH incidence and severity (97).

### 2.4 Management of NAFLD in PCOS

As mentioned, women with PCOS and NAFLD have an elevated fat mass, increased prevalence of hypertrophic obesity, androgen imbalance, lipid disorders, IR, inflammation, OS, HTN, Mets and cardiovascular disorders. The main finding that differentiates the characteristics of PCOS plus NAFLD women from NAFLD patients might be related to HA, as PCOS women with HA experience a higher risk of NAFLD in comparison to PCOS patients without HA, even after adjusting for other potential confounders that have been mentioned above (20).

**Figure 3:**
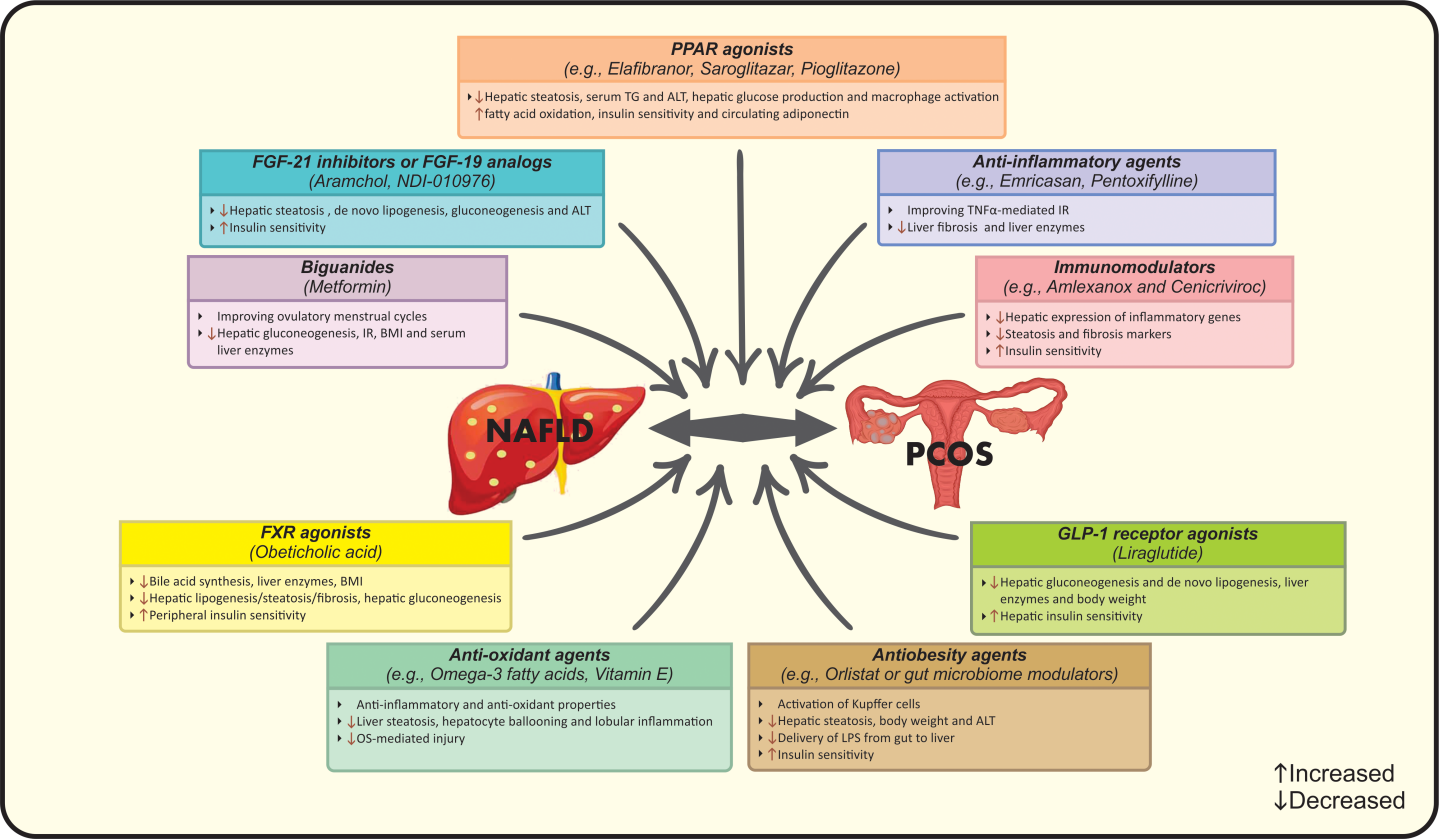
Suggested pharmacological treatments for management of NAFLD in PCOS. ALT, Alanine transaminase; BMI, Body mass index; FGF, Fibroblast growth factor; FXR, Farnesoid X receptor; GLP-1, Glucagon-like peptide 1; IR, Insulin resistance; LPS, Lipopolysaccharides; NAFLD, Nonalcoholic fatty liver disease; OS, Oxidative stress; PCOS, Polycystic ovarian syndrome; PPAR, Peroxisome proliferator-activated receptor; TG, Triglycerides; TNFα, Tumor necrosis factor-alpha.

Applying powerful diagnostic approaches for NAFLD among PCOS women seems necessary due to; firstly, preventing patients from more severe forms of liver disease by considering the higher risk of NASH progression among them and secondly, considering the benefits of early intervention in eliminating NAFLD and related hepatic/metabolic complications (26, 39). NAFLD has been reported to be present in up to 39% of lean PCOS subjects, it is suggested that PCOS patients should undergo NAFLD screening regardless of BMI status or HA (12). A number of diagnostic strategies have been implemented for NAFLD, however the optimal approach is still unclear. Ultrasonography as the first-line screening approach as well as transient elastography (Fibro scan) can be combined with non-invasive liver markers such as serum liver enzyme levels, NAFLD fibrosis score or Fibrosis-4 score to identify NAFLD in PCOS women (98). The combination of ultrasound and serum aminotransferases is commonly used for detection, while liver enzymes cannot be sufficient solely (99). Liver biopsy and MRS are not used in routine practice (99). In addition, PCOS patients should be screened for hepatic and cardio-metabolic disorders. In this context, early diagnosis and managing a useful lifestyle intervention seems necessary (13). The first-line therapeutic strategy for PCOS patients with NAFLD should focus on lifestyle modification including calorie-restricted diet plus regular physical activity resulting in a weigh loss of 5-10% of initial body weight (11, 17, 100). A second-line strategy may also focus on lifestyle modification plus pharmacological therapy (14). Aforementioned interventions aim to improve biochemical, histological and clinical features of both conditions simultaneously (17, 100). Pharmacotherapy is an additional therapeutic approach combined with first-line interventions in a proportion of cases (13). In this context, metformin might be the drug of choice for patients with PCOS and NAFLD (100). It is reported that metformin acts through inhibiting hepatic gluconeogenesis, relieving IR, reducing BMI, improving serum liver enzymes and ovulatory menstrual cycles, but has no effect on liver histology (13, 101). Although PPAR agonists, specially pioglitazone, have shown beneficial effects on IR, elevating adiponectin in circulation, lowering serum ALT, TG and macrophage activation (102, 103). Moreover, the prevalence of NAFLD was reduced in approximately 68% of patients after treatment with liraglutide as a member of glucagon-like peptide-1 (GLP-1) receptor agonists (104). The mechanism of action may be related to increased hepatic insulin sensitivity as well as reduced lipogenesis and liver enzymes (105). Noticeably, evidence regarding the effects of anti-androgenic drugs such as oral contraceptive pills (OCP) on liver markers is still unclear (106). Farnesoid X receptor (FXR) agonists such as obeticholic acid act by reducing bile acid synthesis, liver enzymes, BMI and improving liver steatosis/fibrosis (105). Indeed, FGF-21 inhibitors, FGF-19 analogs and antiobesity agents like aramchol, orlistat or gut microbiome modulators are other therapeutic options for NAFLD treatment that ameliorate liver damage, insulin sensitivity, body weight and the activation of Kupffer cells (105). In the case of gut microbiome modulators, reduced delivery of lipopolysaccharides (LPS) from gut to liver results in treatment (105). Pioneering studies have confirmed the effects of anti-oxidant, anti-inflammatory and immunomodulator agents on PCOS and NAFLD. Emricasan and pentoxifylline have been used as anti-TNF-α medications that alleviate liver enzymes and fibrosis (105). However, amlexanox and cenicriviroc have been examined trough their immune-modulatory activities resulting in down-regulated liver inflammatory genes, steatosis markers as well as IR (105). Omega-3 fatty acids and vitamin E have shown favorable effects on OS- mediated injury, hepatocyte ballooning and lobular inflammation that eventually improve liver steatosis, but further studies are needed to evaluate their clinical effectiveness (107, 108). Other pharmacologic agents e.g. statins, aramchol, anti-fibrotics and probiotics could be used as suggested approaches for NAFLD treatment in PCOS women (10, 17). Suggested pharmacological treatments for management of NAFLD in PCOS are summarized in **Figure 3**. In addition to the aforementioned pharmacotherapies, further investigations are needed to identify the best approach in this context based on the pathological interactions and subsequent complications in both diseases.

### 2.5 Future directions and conclusion

Further studies are needed to investigate other comorbidities associated with NAFLD in PCOS women including cardiometabolic risk factors, gut-liver axis dysfunction, gut microbiota and cardiovascular disease due to the increased risk of mentioned conditions in patients with either disease. Furthermore, larger well- designed cohort studies including periodic assessment of liver function based on biochemical, histological and hormone markers are necessary to explore the current epi-phenomenon with an emphasis on FAI and SHBG. By considering the limitations of previous meta-analysis studies (small sample size, limitations in search strategy and underlying risk factors) conducting more detailed compensatory meta-analysis studies seems desirable. The mechanisms underlying mitochondrial dysfunction during either disease or both together must be noticed. Indeed, improvement of mitochondrial function and endocannabinoid system can be studied as new therapeutic targets. Finally, the most effective, accurate, and evidence-based approaches for the treatment of NAFLD in PCOS women remain unknown, multi-disciplinary research seems to shed light on this issue. NAFLD and PCOS are known as the hepatic and ovarian manifestations of Mets, respectively. In conclusion, the review of literature suggests the significant higher risk of PCOS in NAFLD. The underlying responsible mechanistic pathways are not completely explored. Further research is needed to evaluate the current issue from the “hepato-ovarian axis” to epidemiological, clinical and pharmacological aspects.

## 3 Hypopituitarism

**Figure 4:**
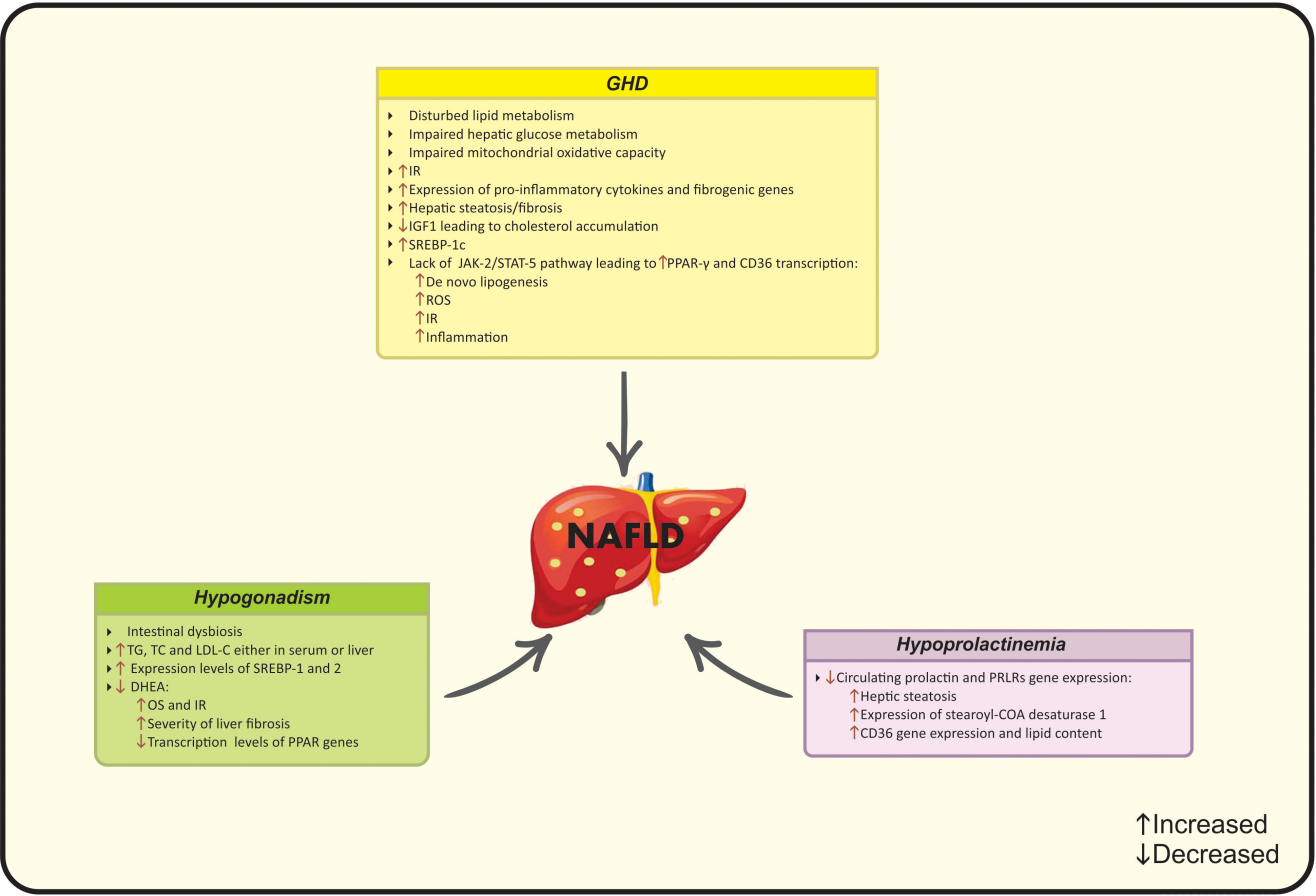
Possible mechanisms of hypopituitarism on NAFLD development. CD36, Cluster of differentiation 36; COA, Coenzyme A; DHEA, Dehydroepiandrosterone; IGF-1, Insulin-like growth factor-1, IR, Insulin resistance; JAK2, Janus kinase 2; LDL-C, Low-density lipoprotein cholesterol; NAFLD, Nonalcoholic fatty liver disease; OS, Oxidative stress; PCOS, Polycystic ovarian syndrome; PPAR, Peroxisome proliferator-activated receptor; PRLR; Prolactin receptor; ROS, Reactive oxygen species; SREBP, Sterol regulatory element- binding protein; STAT-5, Signal transducer and the activation of activator of transcription 5; TC, Total cholesterol; TG, Triglycerides.

The total or partial loss of pituitary gland function caused by pituitary or hypothalamic disorders is defined as hypopituitarism (109). Pituitary gland contributes to the secretion of predominant hormones targeting various organs, including growth hormone (GH), thyroid-stimulating hormone (TSH), gonadotropins and prolactin (110). Hypopituitarism is classified into “primary” and “secondary” subgroups that refer to the internal pituitary gland disorders and other related disorders (hypothalamic and central nervous system), respectively (111). The global prevalence of hypopituitarism ranges from 290 to 455 cases per million and the current incidence is 42.1 cases per million (112). Despite the relative scarcity of hypopituitarism, it is reported that Mets and NAFLD are common conditions that have been observed in these patients (110). More precisely, subjects with hypopituitarism have a potential risk for NAFLD due to the interplay of low GH levels and hypogonadism in the pathogenesis of NAFLD (113). Leptin resistance is another common condition in NAFLD and hypopituitarism and leptin levels correlate with the severity of liver fibrosis in surgical-hypopituitarism patients (114). The main pathophysiological mechanisms linking hypopituitarism to NAFLD are summarized in **Figure 4**.

### 3.1 Growth hormone deficiency

GH and insulin-like growth factor-1 (IGF-1) are mainly mediated by “hypothalamic-pituitary somatotropic axis” and exert several physiological effects, ranging from promoting cell division/proliferation and growth development to macro-nutrients metabolism in adipocytes, liver and skeletal muscle through direct or indirect-complex mechanisms (115, 116). GH also stimulates protein synthesis and lipolysis, hepatic gluconeogenesis and glycogenolysis beside eliminating peripheral insulin sensitivity (117). GHD -a rare clinical syndrome- is resulted from genetic or structural disorders and occurs in both children and adults (110). GHD is defined by insufficient GH secretion from the pituitary gland and is associated with disturbed glucose and lipid metabolism, hypertension and cardiac dysfunction (113, 118).

#### 3.1.1 Epidemiology of NAFLD in GHD

A number of epidemiological studies have demonstrated that GHD patients exhibit higher risk of NAFLD progression, and a negative correlation between GH levels and the severity of NAFLD has been documented. Meanwhile, some studies do not report same results due to the heterogeneity in the characteristics of study participants and sample sizes. The epidemiological studies that have assessed GH and related risk factors in NAFLD patients have been summarized in **Table 2**. Of note, a retrospective study reported a 2% approximate prevalence of NAFLD in patients with hypothalamic/pituitary dysfunctions (120). Additionally, Ichikawa et al. (119) mentioned that patients with GHD exhibit a higher risk for the development of NAFLD, compared to other patients with pituitary dysfunction and the possible association between GHD and NAFLD was suggested based on the “Mets-like phenotype” of adults with GHD (121). Subsequently, cumulative evidence confirmed the aforementioned association in several studies by assessing transaminases (135), ultrasound (122, 123, 127, 132) and biopsy findings (121, 126, 128). Nishizawa et al. (126) reported a 6.4-fold increased prevalence of NAFLD and NASH-related histological findings in subjects with GHD in comparison to matched controls. It should be noted that hypothalamic/pituitary disorders, especially GHD are the risk factors for the secondary forms of NAFLD that are not reversible even after lifestyle modifications (125, 136).

**Table 2:** Studies investigating the risk factors for the development of NAFLD in GHD patients.

Studies	Study design	Study population	Diagnostic criteria	Findings
Ichikawa et al (2003) (119)	Cross-sectional	18 adult patients with hypopituitarism (n=18) GHD patients (n=13) (BMI <25 Kg/m^2^)	NAFLD: computer tomography (liver/spleen CT value <0.9)	Higher prevalence of hepatic steatosis in GHD patients vs hypopituitaric subjects without GHD
Adams et al (2004) (120)	Longitudinal cohort	Patients with pituitary/ hypothalamic dysfunction (n=879) (Mean age: 30 yr., BMI> 25 Kg/m^2^)	NAFLD: Imaging + liver enzyme Alteration, liver biopsy on (n=10) NAFLD patients	GHD patients with NAFLD (n=21) with a 2.3% prevalence
Ichikawa et al (2007) (121)	Cross-sectional	Country: Japan NAFLD patients (n=55) (Mean age: 49.7 yr., Mean BMI: 29 Kg/m^2^)	NAFLD: ALT, ultrasound, CT and liver biopsy	Probable association between low IGF-1 levels and liver fibrosis as well as low GH levels and steatosis
Arturi et al (2011) (122)	Ambulatory-care cross-sectional	**Country: Italy** **Non- diabetic subject**s (n=503) (Mean age: 52 yr., BMI> 25 Kg/m^2^)	**NAFLD: ultrasound**	**Reduced IGF-1 levels in NAFLD patients**
Hong et al (2011) (123)	Cross-sectional observational	Country: Korea Patients with hypopituitarism (n=34) Matched lean healthy controls (n=40) (Mean age: 55 yr., Mean BMI: 25.2 Kg/m^2^)	NAFLD: ultrasound GHD: peak GH level of <3 ng/mL	Significantly higher prevalence of NAFLD in men with hypopituitarism vs controls, lower GH levels in NAFLD patients, negative correlation between GH levels and the severity of steatosis
Koehler et al (2012) (124)	Cross-sectional	Country: USA Patients with complicated Class III obesity (n=160) (Mean age:47.7 yr., Mean BMI: 46.8 Kg/m^2^)	NAFLD and NASH: Liver biopsy	Lower serum levels of GH in obese patients with NASH and advanced fibrosis
Gardner et al (2012) (125)	Cross-sectional	Country: UK GHD patients (n=28) Matched controls (n=24) GHRT group (n=12) (Mean age: 52.6 yr., Mean BMI: 27.8 Kg/m^2^)	NAFLD: MRI-assessed IHLC (IHLC) > 5.6% GHD: GH response <3 mg/L after glucagon stimulation	No differences in liver enzyme and intra-hepatic fat content in GHD group vs controls, reduced adiposity and IHLC with GHRT in patients with baseline high liver fat
Nishizawa et al (2012) (126)	Cross-sectional retrospective	Country: Japan Adults with hypopituitarism and GHD (n=66) Matched healthy controls (n= 83) GHRT group (n=19) (Mean age: 48 yr., Mean BMI: 25 Kg/m^2^)	NAFLD: ultrasound Liver biopsy on (n=16) patients GHD: insulin tolerance test or GH releasing peptide-2 test	Significant higher prevalence (6.4-fold) of NAFLD in GHD group vs controls independently of obesity, higher risk of IR and overweight in GHD+ NAFLD group
Xu et al (2012) (127)	Cross-sectional	Country: China NAFLD patients (n=1667) Healthy controls (n=5479) (Mean age: 49 yr., BMI> 22 Kg/m^2^)	NAFLD: ultrasound	Significant association between GH and NAFLD, GH levels as a risk factor for NAFLD (OR = 0.651)
Sumida et al (2015) (128)	Cross- sectional	Country: Japan Biopsy- proven NAFLD patients (n=199) Matched healthy controls (n=2911) (Age >50 yr., BMI >26 Kg/m^2^)	NAFLD: liver biopsy	Significant lower circulating IGF-1 levels in NAFLD patients vs controls, decreased IGF-1 SDS values by elevated lobular inflammation
Meienberg et al (2016) (129)	Cross-sectional	Multi-ethnic Adult GHD patients (n=22) Matched healthy controls (n=44) GHRT group (n=9) (Mean age: 21.6 yr., Mean BMI: 27.9 Kg/m^2^)	NAFLD: proton magnetic resonance spectroscopy (IHLC >5.56%) GHD: GH levels <7.8 mU/L after glucagon stimulation test	No differences in the prevalence of steatosis and IHLC between two groups, no observed changes in IHLC in GHRT group
Chishima et al (2017) (130)	Cross- sectional	Biopsy-proven NAFLD patients (n=222) Patients with HCV-related- CLD (n=55) (Mean age: 53 yr., Mean BMI: 26.9 Kg/m^2^)	NAFLD: liver biopsy	The role of increased GH levels and lowered IGF-1 levels in NAFLD progression
Dichtel et al (2017) (131)	Retrospective cross- sectional	Multi- racial NAFLD patients (n=142) (Mean age: 50 yr., BMI> 35 Kg/m^2^)	NAFLD: liver biopsy	Significant association between lower serum IGF-1 levels and the severity of NAFLD
Liang et al (2018) (132)	Cross- sectional	Obese children (n=84) Normal weigh children (n=43) (Mean age: 10.5 yr.)	NAFLD: ALT, ultrasound GH: standard provocative testing	Significant inverse relation between NAFLD and IGF-1 as well as GH response test in children
Nguyen et al (2018) (133)	Cross- sectional	Country: France Patients with PD (n=89) Healthy controls (n=74) (Mean age; 53.1 yr., Mean BMI: 29.2 Kg/m^2^)	Steatosis: MRI (LFC>5.5%)	Significant higher prevalence of steatosis and higher LFC in patients with PD due to the lower IGF-1 levels
Yuan et al (2019) (134)	Cross-sectional retrospective	Country: China Adults with hypopituitarism/pan- hypopituitarism (n=50) GHD patients (n=43) (Mean age: 22.8 yr., Mean BMI: 22.2 Kg/m^2^)	NAFLD: ultrasound and liver biopsy GHD: serum GH level<3 μg/L	GHD in 87% of NAFLD patients, no significant differences in serum GH and IGF-1 among studied groups

ALT, Alanine transaminase; GH, growth hormone; GHD, growth hormone deficiency; GHRT, growth hormone replacement therapy; HCV-related- CLD, Hepatitis C virus (HCV)- related chronic liver disease (CLD); IGF-1, Insulin- like growth factor-1; IHLC, intra-hepatic lipid content; IR, Insulin resistance; LFC, Liver fat content; MRI, Magnetic resonance imaging; NAFLD, Non-alcoholic fatty liver disease; NASH, Non-alcoholic steatohepatitis; OR, Odds ratio; PD, Pituitary disease; SDS, Standard deviation score.

Recently, Xu et al. (127) conducted a large cross-sectional study reporting a significant relation between GH levels and the risk factors of NAFLD (OR: 0.651). Lower GH and IGF-1 levels are correlated with the severity of NAFLD (122, 127, 128, 130–133, 137). In this regard, mRNA levels of hepatic IGF-1 obviously decreases in NASH patients in comparison to patients with liver steatosis and predicts the degrees of hepatic inflammation (138). Moreover, the current body of evidence suggests that NAFLD/NASH patients, even with advanced liver fibrosis, have low serum GH levels (123, 124, 128, 132). Hence, NAFLD might be known as a hepatic complication of GDH. Despite the agreement among the majority of studies, Yuan et al. (134) have not found any differences in GH and IGF-1 levels between NAFLD patients and healthy controls. Furthermore, another study reported similar prevalence of NAFLD through magnetic resonance imaging (MRI)-assessment between GHD patients and healthy controls (129).

#### 3.1.2 Pathophysiology of NAFLD in GHD

GHD is followed by a wide range of metabolic consequences that are linked to the onset of NAFLD, including visceral obesity, IR and dyslipidemia (139). Beside the direct effects of GH, it could act indirectly through IGF-1 production mainly by the liver (117). IGF-1 amplifies GH actions after binding to specific proteins and receptors (117). In this regard, GH/IGF-1 axis is involved in the progression of NAFLD and predicts liver steatosis/fibrosis. Moreover, based on the evidence from animal studies, lower IGF-1 levels are associated with lower protection against IR, inflammation and liver steatosis, while, treatment with IGF-1 improves liver enzymes, steatosis and fibrosis (115). Impaired hepatic GH signaling is immediately followed by liver steatosis and NASH that highlights the role of GH in the liver in an independent manner from metabolic dysfunction (113).

##### 3.1.2.1 Insulin resistance

There is an inverse interaction between GH and insulin signaling *via* the stimulation of hepatic gluconeogenesis and glycogenolysis. However, GHD is along with IR and impaired hepatic glucose metabolism due to the changes in lipid flux (140).

##### 3.1.2.2 Inflammation

GHD up-regulates the expression levels of adipocytes and pro-inflammatory cytokines that leads to increased hepatic and visceral fat accumulation and IR (139). Elevated expression of inflammatory/fibrogenic genes including TNFα and chemokine (C-C motif) ligand 3 (CCL3) are observed in animals with liver GH receptor deletion (141). This systemic low-grade inflammation also stimulates the progression of NAFLD in GHD patients. IGF-1 acts as an anti-inflammatory and anti-oxidant agent by suppressing ROS generation and promoting hepatic mitochondrial function (126). Moreover, GH replacement improves inflammatory state in human (14).

##### 3.1.2.3 Oxidative stress

Current evidence suggest the protective role of GH against OS by regulating mitochondrial oxidative capacity. OS is a key pathologic hit in the progression of NAFLD. As discussed, lower IGF-1 levels increase OS and induce NAFLD that suggests IGF-1 as an anti-oxidant factor (142). Recent studies have investigated an association between IGF-1 and sirtuin-4 (Sirt4) levels (143). Sirt4- a mitochondrial NAD-dependent ADP ribosyltransferase- negatively regulates the oxidative capacity and lowers levels of free fatty acid and ROS (143).

Melatonin is another contributing factor involved in the epi-phenomenon. Melatonin as a pleiotropic hormone act as a ROS-scavenger and exerts ant-oxidative activity (144). During NAFLD, melatonin improves oxidative state and liver histology (144). Notably, melatonin could modulate the secretion of GH and exert beneficial effects on GHD and NAFLD in the same time (145).

#### 3.1.3 Mechanisms linking NAFLD to GHD

The correlation of GH and IGF-1 with NAFLD has been reported in epidemiological studies. IGF-1 mediates lipid metabolism *via* several intracellular pathways. IGF-1 up-regulates the expression of ATP-binding cassette transporter A1 (ABCA1) and prevents cholesterol accumulation (146). Taken together, based on the changes in liver homeostasis, function and structure due to impaired GH/IGF-1 axis, treatment with IGF-1 induces the senescence of the liver stellate cells and improves hepatic fibrosis (147). Additionally, the involvement of GH/IGF-1 axis in lipid metabolism is also observed through regulating Janus kinase 2 (JAK-2) and signal transducer and the activation of activator of transcription 5 (STAT-5) (148). GHD is along with severe hepatic steatosis, fibrosis, adipogenesis, and IR due to the lack of aforementioned genes (139). GH stimulates JAK2-STAT5 activation, promotes lipolysis and insulin sensitivity and attenuates lipogenesis (149). The activation of STAT-5b may inhibit the expression of downstream genes. In this context, GH inhibits PPAR-γ and Cluster of differentiation 36 (CD36) transcription as the chief regulators of free fatty acid uptake and attenuates *de novo* lipogenesis (150). PPAR-γ/CD36 are lipid activating factors involved in the ROS production in NAFLD (151). PPAR-γ stimulates the expression of genes and production of enzymes related to lipid metabolism and CD36 facilitates lipid endocytosis (152, 153). Higher CD36 production aggravates IR and inflammation. GH reduces the production of CD36, and the deletion of CD36 increases leptin secretion and suppresses lipid absorption (153). Sterol regulatory element- binding protein-1c (SREBP-1c) is a transcription factor involved in both conditions that regulates hepatic adipogenesis and induces lipid storage (154). Suppressed expression of SREBP-1c eliminates the risk of NAFLD, while, SREBP-1c is up-regulated in GHD diseases (139). GH administration interacts with this condition and improves lipid status as well as body weight (155).

#### 3.1.4 Management of NAFLD in GHD

Several tests are implemented in clinical practice with the aim of GHD diagnosis including insulin tolerance test and GH-releasing hormone (GHRH)-arginine test (14). Although there is not any definite identified cut-off value for GHD, ranges of <5.1 g/L for the insulin tolerance test and 4.1 g/L for the GHRH-arginine test suggest the state of GHD (143). GH replacement therapy has been used in a number of studies exerting improvements in transaminase levels, inflammation, mitochondrial function, lipid profile, liver steatosis/fibrosis and weight reduction (14, 113). GH replacement therapy reduces serum TNF-α, OS and other NAFLD-related conditions after six-month intervention (143). Moreover, recombinant human GH has been applied in adolescents with NAFLD (156). Collectively, despite the mentioned favorable effects, the efficacy of GH-replacement therapy (GHRT) in GHD patients with NAFLD is still controversial. Combining lifestyle intervention to specific therapy seems to be more effective for the treatment, but not sufficient for the prevention of NAFLD in these patients (14).

#### 3.1.5 Future directions and conclusion

In summary, NAFLD screening is needed in all patients with GHD. Despite the controversy among recent finding, treatment with GH or IGF-1 might be a useful strategy in NAFLD patients with GHD. However, subsequent well-designed studies can evaluate the efficacy of aforementioned therapeutic strategies.

### 3.2 Hypogonadism

As shown before, metabolic disturbances are parallel to hormonal abnormalities, triggering each other. According to the “sex hormone-liver axis”, male hypogonadism and female hyperandrogenism are recently known as the independent risk factors for the development of NAFLD (157). The diminished function/activity of the reproductive organs followed by reduced sex hormone concentrations in either sex and regardless of its pathological causes is defined as hypogonadism and is classified into primary and secondary subtypes. Secondary hypogonadism is related to the reduced hypothalamus/pituitary gland function in producing sex hormones (158). The basic diagnostic criteria include the combination of clinical and biochemical signs of low sex hormones beside changes in physical health. Moreover, considering the co-existence of hypogonadism along with other endocrinopathies, such as hypopituitarism may be helpful (159). NAFLD is a common observed condition in these patients and shares a bi-directional connection with hypogonadism.

#### 3.2.1 Epidemiology of NAFLD in hypogonadism

To the best of our knowledge, a great number of studies have investigated the bi-directional relationship between hypogonadism and NAFLD (160–162). Women with hypogonadism and hypoestrogenism exhibit higher prevalence of liver enzyme levels, NAFLD and advanced liver fibrosis (91, 92, 160, 163–168). **Table 3** summarizes the results of studies investigating the potent association between hypogonadism and NAFLD in men and women. As discussed, increased duration of estrogen deficiency has been reported to be associated with an increased risk of hepatic fibrosis (160). Mckenzie et al. (165) reported that hormone replacement therapy (HRT) alleviates serum liver enzymes in diabetic post-menopausal women.

**Table 3:** Studies investigating the risk of NAFLD in Hypogonadism.

Studies	Study design	Study population	Findings
Elsheikh et al (2001) (163)	Prospective study	Women with TS (n=80) TS women receiving HRT (n=20) (Mean age: 26 yr., Mean BMI: 24.6 Kg/m^2^)	Increased liver enzymes in 44% of TS women, significant improvements in liver enzymes after HRT
Bruno et al (2005) (164)	Prospective, double-blind, RCT	Country: Italy Women enrolled into the multi-centric Italian tamoxifen chemoprevention trial (n=5408) Women after follow up (n=64) (Age: 35-70 yr., BMI>25 Kg/m^2^)	Higher risk of NASH in overweight/obese receiving tamoxifen as an estrogen receptor modulator
McKenzie et al (2006) (165)	Double-blind, RCT	Country: UK Women with T2DM (n=50) receiving HRT or placebo (Mean age: 60 yr., BMI>25 Kg/m^2^)	Significant reduction in liver enzymes after HRT
Charlton et al (2008) (169)	Cohort	Mild/advanced biopsy-proven NAFLD patients (n=439) (Mean age: 47 yr., BMI>25 Kg/m^2^)	Lower serum DHEA-S in advanced NAFLD vs mild cases, lowered DHEA-S along with the severity of fibrosis in NAFLD
El-Mansoury et al (2008) (167)	Cross-sectional	Country: Sweden Women with TS (n=218) (Mean age: 33 yr., Mean BMI: 24.5 Kg/m^2^)	Increased liver enzymes in 36% of TS women, especially GGT, no effect of estrogen substitute withdrawal on serum liver enzymes.
Koulouri et al (2008) (166)	Cross-sectional	Country: UK Women receiving HRT (n=169) Women with TS (n=14) Hypogonadic controls (n=11) (Mean age: 31 yr., Mean BMI: 25.8 Kg/m^2^)	An association between higher doses of HRT with lower RLE, TC and BMI, increased prevalence of RLE in TS women (91%)
Gutierrez-Grobe et al (2010) (91)	Cross-sectional	Women with NAFLD (n=93) Healthy controls (n=93)	Lower levels of E2 in women with NAFLD
Haider et al (2010) (170)	Cohort	Hypogonadic men treated with HRT (n=117) (Age: 34-69 yr.)	Significant reduction in RLE, steatosis and inflammation after following 1-year HRT
Sumida et al (2010) (171)	Cross-sectional	Country: Japan Biopsy- proven NAFLD patients (n=133) Including NASH (n=90) Fibrosis (n=73) Advances fibrosis (n=17) Healthy controls (n=399) (Mean age: 55 yr., varied BMI)	Lower DHEA-S levels along with more severe grades of liver fibrosis
Koga et al (2011) (172)	Cross-sectional	Country: Japan Men with NAFLD (n=69) (Mean age: 50 yr., varied BMI)	Positive correlation between serum ALT and DHEA-S levels, increased DHEA-S levels in NAFLD patients
Koehler et al (2012) (124)	Cross-sectional	Multi- racial Morbidly obese patients underwent biopsy (n=160) Patients with steatosis (n=72) NASH with fibrosis (stage 0-1) (n=60) NASH with fibrosis (higher stages) (n=12) Healthy liver (n=16) (Mean age: 47.7 yr., Mean BMI: 46.8 Kg/m^2^)	Lower DHEA serum levels in obese cases with NASH and fibrosis
Tokushige et al (2013) (173)	Cohort	Country: Japan Biopsy- proven NAFLD (training cohort) (n=44) Biopsy- proven NAFLD (validation cohort) (n=105) Biopsy- proven PBC patients (n=26) Healthy controls (n=48) (Mean age: 55.8 yr.)	An association between DHEA-S levels and hepatic fibrosis just in NAFLD patients and not in PBC group
Yang et al (2014) (162)	Cross- sectional	Biopsy-proven NASH patients (n=541) (Mean age: 49.3 yr., Mean BMI: 23.1 Kg/m^2^)	Higher risk of liver fibrosis in men and post-menopausal women due to the protective role of estrogen
Hanew et al (2016) (168)	Cross- sectional	Country: Japan Patients with TS (n=492) (Mean age: 26.6 yr., varied BMI)	Higher prevalence of DM, HTN, dyslipidemia, and liver dysfunction in TS women and related to increasing BMI
Klair et al (2016) (160)	Cross-sectional	Multi- ethnic Post-menopausal women with NAFLD (n=488) (Age >55 yr., BMI >30 Kg/m^2^)	Association between duration of estrogen deficiency and fibrosis risk among post-menopausal women with NAFLD
Wang et al (2016) (174)	Retrospective case-control	Country: China Men with IHH (n=75) Men with IHH and NAFLD (n=63) Healthy controls (n=135) (Mean age: 21 yr., varied BMI)	Significant higher DHEA-S levels in IHH patients with NAFLD vs without NAFLD
Yang et al (2016) (162)	Retrospective study	Country: Korea Women receiving SERM treatment for breast cancer (n=1061) (Mean age: 49.3 yr., Mean BMI: 23.1 Kg/m^2^)	Higher risk of NAFLD or RLE after SERM administration
Jaruvongvanich et al (2017) (175)	Meta- analysis	16 trials including men (n=13,721) and women (n=5,840) (Age >15 yr., varied BMI)	Positive association between lower TT levels and NAFLD in men and negative association in women, negative association between SHBG levels and NAFLD odds in both genders
Gild et al (2018) (161)	Cross-sectional	Multi- racial Elderly men with prostate cancer (n=380,669) Elderly men receiving ADT (n=31,117) (Mean age: 21.6 yr)	Higher chance of NAFLD, cirrhosis and liver disease in ADT administrated patients
Polyzos et al (2020) (176)	Cross-sectional	Country: Greece Men (n=98) (Mean age: 36 yr., BMI >25 Kg/m^2^)	Negative association between TT levels and NAFLD- related indices, except liver fibrosis indices

ADT, androgen deprivation therapy; ALT, Alanine transaminase; BMI, body mass index; DHEA-S, sulfated dehydroepiandrosterone; E2, 17-β-estradiol; GGT, Gamma-glutamyl transferase; HRT, hormone replacement therapy; HTN, Hypertension; IHH, idiopathic hypogonadotropic hypogonadism; NAFLD, nonalcoholic fatty liver disease; NASH, Non-alcoholic steatohepatitis; PBC, primary biliary cholangitis; RCT, randomized controlled trial; RLE, raised liver enzymes; SERM, selective estrogen receptor modulator; SHBG, Sex hormone binding globulin; T2DM, Type-2 diabetes mellitus; TC, Total cholesterol; TS, Turner syndrome; TT, total testosterone.

Serum TT levels are associated with central fat accumulation, IR and Mets (139). Seo et al. (177) in a cross-sectional study on 1944 men, reported lower TT levels in NAFLD patients in comparison to controls. Indeed, other studies confirmed these findings (170, 178, 179). Haider et al. (170) reported the favorable effects of testosterone replacement therapy on liver function of male NAFLD subjects. Additionally, the higher risk of NAFLD after androgen deprivation therapy in men with prostate cancer may be eliminated through hormone replacement therapy (HRT) (161). Jaruvongvanich et al. (175) performed a meta-analysis on 16 observational studies (13,721 men) and documented that TT levels are lower in NAFLD men in comparison to controls. Based on the sex-dependent relation, TT levels have inverse-association with NAFLD in men and positive-association in women, while, higher SHBG levels are followed by lower NAFLD odds in either gender (175).

In this regard, the same association has been observed in children and adolescent, as Mueller et al. (180) in a cross-sectional multiethnic study reported that boys with increased TT had lower steatosis/fibrosis in histological findings, while girls with higher TT experienced more severe forms of NAFLD and approved the sex-dependent association of TT levels with NAFLD even in pediatric population. Polyzos et al. (176) observed a negative-relationship between TT levels and steatosis-related indices, but not with fibrosis indices.

In addition, some studies have investigated the relationship between lower dehydroepiandrosterone (DHEA) levels and liver-related complications in NASH patients and have suggested DHEA as a marker for assessing the risk of NASH with advanced fibrosis (14).

#### 3.2.2 Pathophysiology of NAFLD in hypogonadism

The exact underlying mechanism in the incidence of “disturbed sex hormone- liver axis” state remains unclear, while deregulated pancreatic β-cell, adipocyte and hepatocyte function and changes in energy homeostasis could be considered as indirect changes during hormonal imbalances (181). Sex hormone deficiency is associated with disruption of homeostasis of lipid and glucose metabolism, triggering the molecular pathways leading to NAFLD (14). Although, detecting the exact molecular mediators is very complex, a number of common underlying pathogenic pathways are involved.

##### 3.2.2.1 Female hypogonadism

Decreases in sex steroids are observed in women during menopause (158). Men and post-menopause women experience higher risk of Mets and NAFLD compared with pre-menopause women (182). This higher risk reaches to approximately two-fold higher risk of NAFLD in post-menopausal women (139). The underlying mechanism is the effect of reduced estrogen levels on increasing hepatic fat accumulation, dyslipidemia and the incidence of NAFLD (158). Estrogen also improves anti-oxidant state and protects women from liver injury. Furthermore, increased testosterone and decreased SHBG levels are associated with NAFLD in post- menopause women (183, 184).

Hypogonadism in females is commonly resulted from Turner syndrome-defined by premature ovarian failure and subsequent hypoestrogenism- that eventually leads to elevated hepatic enzymes, NAFLD and liver injury (185). In addition, female rats have shown development of NAFLD due to the lowered fibroblast growth factor (FGF)-21 after ovariectomy (186).

##### 3.2.2.2 Male hypogonadism

Low testosterone levels are observed in male hypogonadism. Low testosterone levels are correlated with IR in a bi-directional manner followed by abdominal/visceral fat deposition (187). In turn, adipocytes generate leptin and pro-inflammatory factors that inhibit the production of testosterone. Taken together, epidemiologic studies mention that lower testosterone levels are correlated with the prevalence of NAFLD (178, 179).

#### 3.2.3 Mechanisms linking NAFLD to hypogonadism

Sex steroid deficiency is mainly involved in the pathogenesis of NAFLD as estrogen and testosterone play role in liver steatosis. Moreover, a number of other pathologic changes also occur during hypogonadal state.

##### 3.2.3.1 Estrogen

Estrogen regulates energy and lipid homeostasis through its classic nuclear estrogen receptors (ERs) in the liver, exerts non-nuclear activities and inhibits liver fibrosis (64, 143). Estrogen reduces hepatic lipid deposition and promotes fatty acid oxidation *via* inhibiting PPAR-α signaling pathway (139). Indeed, increases the expression levels of insulin receptor gene. Therefor, hypoestrogenism triggers IR and disturbed glucose tolerance (188). Lower estradiol levels are also correlated with hepatic fat accumulation (189). Moreover, hypoestrogenism is associated with increased macrophage infiltration and higher expression of pro-inflammatory genes in the liver (190).

##### 3.2.3.2 Androgens

The major circulating androgens-testosterone and dihydrotestosterone (DHT)-affect hepatic metabolism. Testosterone modulates gene expression by translocating to the nucleus after binding to the hepatic ARs in both male and females (158). The highest expression levels of ARs is attributed to males and subjects in reproductive years (64). The polymorphic CAG repeat sequence of AR gene affects fat accumulation (191, 192). Of note, testosterone improves pancreatic function, suppresses TG uptake, adipose accumulation and inhibits adipocyte differentiation (193). Testosterone levels negatively correlate with visceral fat mass and the incidence of obesity and obesity-related conditions including Mets, T2DM and NAFLD in men with hypogonadism (194, 195).

Collectively, observational studies suggest that testosterone levels are associated with NAFLD in a sex- dependent manner, as higher serum TT levels lead to steatosis in women, while may be even protective in men. More precisely, TT levels in men and post-menopause women are negatively correlated with the incidence of NAFLD, whereas the correlation seems positive in PCOS women (157, 175). In the case of SHBG, diminished SHBG levels are associated with NAFLD in either sex (183).

##### 3.2.3.3 Dehydroepiandrosterone

DHEA deficiency is commonly observed in pan-hypopituitarism condition and amplifies the rapid progression of NASH to cirrhosis. DHEA is a steroid hormone with high abundance levels and modulates homeostasis, mediates ROS scavengers and OS, reduces IR, and increases the transcription levels of PPAR genes (169, 196). Several studies have illustrated the association between diminished serum DHEA levels and the higher severity of liver fibrosis in biopsy-proven NASH patients (124, 169, 171–174, 177). In this regard, the settled studies have been summarized in **Table 3**.

##### 3.2.3.4 Cardiovascular factors

Hypogonadism triggers a wide range of impairments in cardiovascular risk factors. The pattern of fat distribution as well as regional adiposity is dependent on sex hormones and hypogonadism which causes obesity, dyslipidemia, hypertension and dysglycemia and resulting in the higher progression of NAFLD (14). Based on former studies, animals in hypogonadal state experience increased TG, total cholesterol and LDL-C either in serum or liver and show elevated expression levels of SREBP-1 and 2 that eventually enhance liver steatosis (197).

##### 3.2.3.5 Gut microbiota

Based on the evidence from animal studies, intestinal dysbiosis links hypogonadism to the development of NAFLD. Changes in Lactobacillus numbers as well as Firmicutes/Bacteroidetes ratio could be resulted from androgen loss in castrated male mice (198).

#### 3.2.4 Management of NAFLD in hypogonadism

Patients with hypogonadism-induced NAFLD should regularly be visited by both endocrinologist and hepatologist and undergo liver related assessments after excluding other steatogenic causes to prevent liver damage (199). The general management includes a combination of lifestyle modifications, dietary restrictions as well as regular physical activity (200). Specific treatment plans should be sex-dependent, i.e. female hypogonadism is treated by estrogen replacement, while in men with hypogonadism testosterone is the drug of choice (14, 201).

#### 3.2.5 Future directions and conclusion

Further studies evaluating the potential impact of HRT in patients with hypogonadism and NAFLD are needed. As mentioned before, hypogonadism is linked to NAFLD in a bi-directional manner and these patients should be screened routinely.

### 3.3 Hypoprolactinemia

Anterior-pituitary is responsible for the production of prolactin- a multifunctional lactotroph polypeptide hormone (202). Hypopituitarism is usually along with reduced levels of prolactin and expression levels of prolactin receptors (PRLRs) gene (139). Prolactin is involved in growth, immunity, food intake and metabolic health *via* modulating glucose and lipid metabolisms as well as stimulating adipogenesis (113). Prolactin is also involved in the biosynthesis of monounsaturated fatty acids by down-regulating the expression of stearoyl-CoA desaturase1 (SCD1) (203). Moreover, it acts through its specific hepatic receptors and down-regulates CD36, resulting in reduced hepatic TG accumulation, lowered lipid content and protection against hepatic steatosis (203, 204). Luque et al. (205) highlighted the probable role of prolactin in *de novo* lipogenesis and subsequent NAFLD progression. Zhang et al. (204) in a study on 456 NAFLD subjects reported lower circulating prolactin levels in NAFLD group in comparison to controls. Prolactin levels were lower in patients with more severe hepatic steatosis (139). In conclusion, although regarding evidence seems to be insufficient and further *in vivo* and *in vitro* studies are needed, chronic lower prolactin levels may exert catastrophic effects on liver health.

## 4 Conclusion

Taken together, the current body of evidence highlights the inter-relationship between NAFLD and a wide range of endocrinopathies and suggests the need of NAFLD screening, simultaneously by hepatologists and endocrinologists. Further well-designed studies are needed to discover the exact underlying molecular contact-point of aforementioned diseases as well as the possible new screening/treatment strategies for NAFLD patients with endocrine disorders.

## Author contributions

SA and HT contributed to data extraction and manuscript drafting. ME-M and FN contributed to the conception of the article as well as to the final revision of the manuscript. All authors read and approved the final version of the manuscript (grant No 71003).

## Funding

This study was supported by the Research Vice-Chancellor and Endocrine Research Center of Tabriz University of Medical Sciences (grant No 71003).

## Conflict of interest

The authors declare that the research was conducted in the absence of any commercial or financial relationships that could be construed as a potential conflict of interest.

## Publisher’s note

All claims expressed in this article are solely those of the authors and do not necessarily represent those of their affiliated organizations, or those of the publisher, the editors and the reviewers. Any product that may be evaluated in this article, or claim that may be made by its manufacturer, is not guaranteed or endorsed by the publisher.

## Glossary

**Table:**

ABCA1	ATP-binding cassette transporter A1
ADT	Androgen deprivation therapy
ALT	Alanine transaminase
AMPK	Adenosine monophosphate-activated protein kinase
APRI	AST to platelet ratio
ARs	Androgen receptors
AST	Aspartate aminotransferase
BMI	Body mass index
CB	Cannabinoids receptor
CCL3	Chemokine (C-C motif) ligand 3
CD36	Cluster of differentiation 36
CK18	Cytokeratin 18
CLD	Chronic liver disease
DHEA-S	Sulfated dehydroepiandrosterone
DHT	Dihydrotestosterone
E2	17-β-estradiol
ECS	Endocannabinoid system
ERs	Estrogen receptors
FAI	Free androgen index
FBS	Fasting blood sugar
FGF-21	Fibroblast growth factor
FLI	fatty liver index
FSH	follicle-stimulating hormone
FXR	Farnesoid X receptor
GGT	gamma- glutamyl transferase
GH	Growth hormone
GHD	Growth hormone deficiency
GHRH	GH-releasing hormone
GHRT	Growth hormone -replacement therapy
GLP-1	Glucagon-like peptide-1
GWAS	Genome-wide association studies
HA	Hyperandrogenism
HCC	Hepatocellular carcinoma
HCV	Hepatitis C virus
HDL	High-density lipoprotein cholesterol
HRT	Hormone replacement therapy
HSI	Hepatic steatosis index
HTN	Hypertension
HOMA-IR	Homeostatic model assessment of insulin resistance
IGF-1	Insulin-like growth factor-1
IHH	Idiopathic hypogonadotropic hypogonadism
IHLC	Intra-hepatic lipid content
IR	Insulin resistance
JAK2	Janus kinase 2
LAP	Lipid accumulation products
LDL	Low-density lipoprotein
LDLR	Low-density lipoprotein receptor
LFC	Liver fat content
LH	Luteinizing hormone
LPS	Lipopolysaccharides
MAFLD	Metabolic dysfunction-associated fatty liver disease
MAP	Mitogen-activated protein
MAPK	Mitogen-activated protein kinase
MEK/ERK	Mitogen-activated protein kinase/extracellular signal-regulated kinase
Mets	Metabolic syndrome
MRI	Magnetic resonance imaging
MRS	Magnetic resonance spectroscopy
NAFLD	Nonalcoholic fatty liver disease
NASH	Non-alcoholic steatohepatitis
NIH	National Institutes of Health
OCP	Oral contraceptive pills
OR	Odds ratio
OS	Oxidative stress
OSA	Obstructive sleep apnea
PBC	Primary biliary cholangitis
PCOS	Polycystic ovarian syndrome
PD	Pituitary disease
PI3K	Phosphoinositide 3-kinase
PNPLA3	Patatin-like phospholipase domain-containing
PPARα	Peroxisome proliferator-activated receptor-α
PRLRs	Prolactin receptors
RCT	Randomized controlled trial
RLE	Raised liver enzymes
ROS	Reactive oxygen species
SCD1	Stearoylcoenzyme A desaturase 1
SDS	Standard deviation scores
SERM	Selective estrogen receptor modulator
SHBG	Sex hormone-binding globulin
Sirt-4	Sirtuin-4
SREBP	Sterol regulatory element- binding protein
STAT-5	Signal transducer and the activation of activator of transcription 5
T2DM	Type 2 diabetes mellitus
TC	Total cholesterol
TG	Triglycerides
TNFα	Tumor necrosis factor-alpha
TS	Turner syndrome
TSH	Thyroid-stimulating hormone
TT	Total testosterone
VLDL	Very low-density lipoprotein cholesterol
